# Climate Change and Range Expansion of the Asian Tiger Mosquito (*Aedes albopictus*) in Northeastern USA: Implications for Public Health Practitioners

**DOI:** 10.1371/journal.pone.0060874

**Published:** 2013-04-02

**Authors:** Ilia Rochlin, Dominick V. Ninivaggi, Michael L. Hutchinson, Ary Farajollahi

**Affiliations:** 1 Suffolk County Vector Control, Yaphank, New York, United States of America; 2 Division of Vector Management, Pennsylvania Department of Environmental Protection, Harrisburg, Pennsylvania, United States of America; 3 Mercer County Mosquito Control, West Trenton, New Jersey, United States of America; 4 Center for Vector Biology, Rutgers University, New Brunswick, New Jersey, United States of America; Universidade Federal do Rio de Janeiro, Brazil

## Abstract

The Asian tiger mosquito, *Aedes albopictus* (Skuse), is an invasive species with substantial biting activity, high disease vector potential, and a global distribution that continues to expand. New Jersey, southern New York, and Pennsylvania are currently the northernmost boundary of established *Ae. albopictus* populations in the eastern United States. Using positive geographic locations from these areas, we modeled the potential future range expansion of *Ae. albopictus* in northeastern USA under two climate change scenarios. The land area with environmental conditions suitable for *Ae. albopictus* populations is expected to increase from the current 5% to 16% in the next two decades and to 43%–49% by the end of the century. Presently, about one-third of the total human population of 55 million in northeastern USA reside in urban areas where *Ae. albopictus* is present. This number is predicted to double to about 60% by the end of the century, encompassing all major urban centers and placing over 30 million people under the threat of dense *Ae. albopictus* infestations. This mosquito species presents unique challenges to public health agencies and has already strained the resources available to mosquito control programs within its current range. As it continues to expand into areas with fewer resources and limited organized mosquito control, these challenges will be further exacerbated. Anticipating areas of potential establishment, while planning ahead and gathering sufficient resources will be the key for successful public health campaigns. A broad effort in community sanitation and education at all levels of government and the private sector will be required until new control techniques are developed that can be applied efficiently and effectively at reasonable cost to very large areas.

## Introduction

Mosquitoes are the single most important taxon of arthropods affecting human health globally [1] and are also amongst the most prolific invasive species contributing to the spread of endemic or exotic diseases [2]. The Asian tiger mosquito, *Aedes albopictus* (Skuse), is a highly invasive container-inhabiting species that has dispersed widely from its native range in Southeast Asia and is now found on all continents but Antarctica [3], [4]. In many parts of its expanded range, this species has been implicated as a significant vector of re-emerging arthropod-borne viruses such as chikungunya, dengue, and West Nile (WNV). The recent outbreaks and reemergence of chikungunya in the Indian Ocean basin were driven primarily by *Ae. albopictus* and attributed to a viral mutation which enhanced the vector competency and transmission efficiency by this species [5]. Autochthonous transmissions of chikungunya in temperate northern Italy and southeastern France [6], [7] and dengue in France and Croatia [8] were made possible by locally established *Ae. albopictus* populations. Similarly, *Ae. albopictus* was implicated in the resurgence of both chikungunya and dengue in Central Africa [9].

In North America, *Ae. albopictus* is among the most efficient bridge vectors of WNV [10]–[12]. In addition to vectoring exotic arboviruses, this species can also transmit the endemic eastern equine encephalitis and La Crosse viruses in the laboratory and in the field [13]–[16] creating a potential for the resurgence of mosquito-borne diseases native to North America [17]. Since this species is commonly associated with human habitation and urbanized environments, high *Ae. albopictus* populations represent an important public health problem in many parts of the world due to severe human biting activity [4], [8].

**Figure 1:**
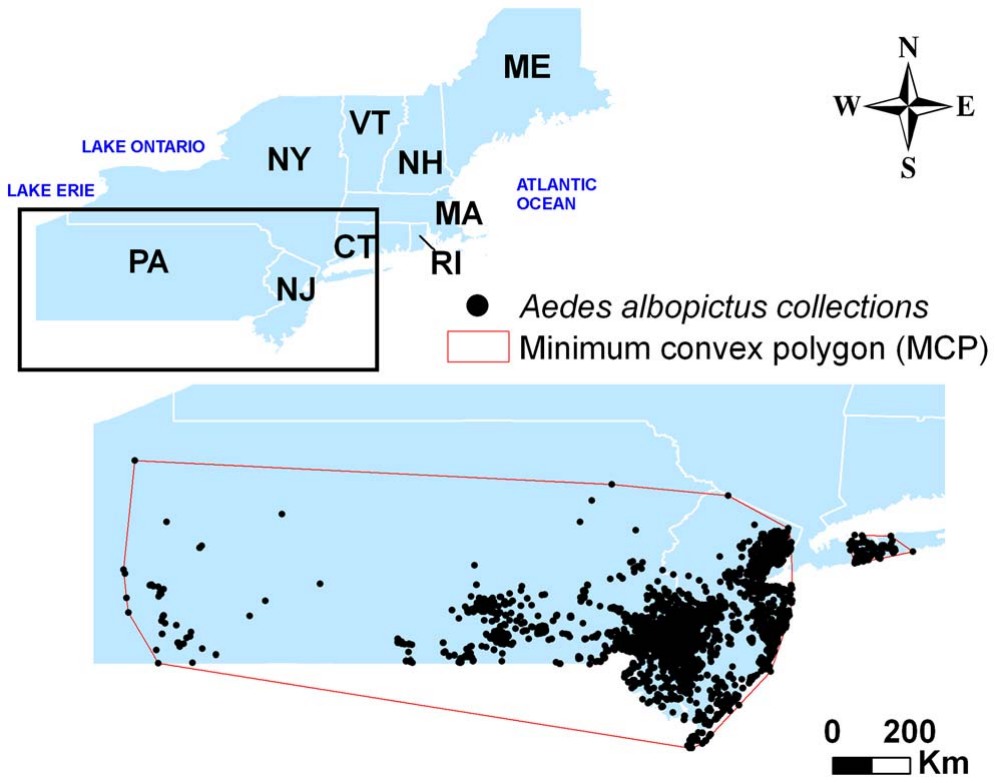
Study area and minimum convex polygon (MCP) around *Ae. albopictus* collection locations delineating general “presence” region for Maxent modeling.

Extraordinary invasion propensities and public health significance of the Asian tiger mosquito have attracted substantial attention in the United States since this species first became established in Texas in 1985 [18]. Following the introduction, *Ae. albopictus* has spread to 36 states and continues to expand its range [4]. Presently, *Ae. albopictus* reaches its northernmost boundary in the northeastern USA with established populations in parts of New Jersey, southern New York (Long Island), and Pennsylvania (Figure 1). Winter temperature likely plays the most important role in arresting its further range expansion northward [19]–[22] with winter precipitation serving as a possible moderating factor [23]. Previous global modeling studies have predicted the extent of this species' range in northeastern USA under current climatic conditions [3], [24]. These approaches have coarse resolution which is less useful for planning on the regional or local level. Moreover, global climate change is expected to affect the future weather patterns in northeastern USA, especially winter temperatures, which are predicted to rise by between 1.7°C to 5.4°C in this century [25]. Thus, this study's goals were (a) to model future expansion of *Ae. albopictus* in northeastern USA based on known geographic locations at the present and future climate projections until 2099, and (b) to discuss the implications for local public health and vector control professionals as *Ae. albopictus* continues to expand its range.

## Materials and Methods

No specific permits were required for the collections of adult mosquitoes, which were conducted with homeowners assent by professional county mosquito control personnel. This study did not involve endangered or protected species.

### Data sources

#### Environmental layers

Climatic and landscape variables used in this study are listed in Table 1. The baseline (1950–2000) temperature and precipitation layers were obtained from WorldClim global climate data repository (www.worldclim.org). Future climatic data integrated two CO_2_ emission scenarios, moderate (B2) and high (A2), detailed in the Special Report on Emissions Scenarios by the Intergovernmental Panel on Climate Change [26]. The climate layers created using CCCma second generation coupled global climate model (CGCM2) were acquired from the International Center for Tropical Agriculture (www.ccafs-climate.org) for three time periods: 2020s (2010–2039), 2050s (2040–2069), and 2080s (2070–2099). Elevation and 2006 Land use/cover (LUC) data were obtained from the WorldClim and the National Landcover Database (www.mrlc.gov), respectively. The 2006 LUC data were reclassified to Level I and resampled at the native WorldClim 30 arcsec (approximately 1×1 km) resolution. The northeastern USA coverage was extracted from the global or national datasets to include the states of Connecticut, Maine, Massachusetts, New Jersey, New York, Pennsylvania, Rhode Island, and Vermont (Figure 1). Urban area information and shapefiles for 2010 Census were acquired from the US Census Bureau (www.census.gov).

**Table 1:** Environmental variables used in the analysis and model selection. Variables included in the final model are indicated in bold.

Variable	Abbreviation	Inclusion in the final model
Annual Mean Temperature	bio1	No, highly correlated with bio11
Mean Diurnal Range	bio2	No, not significant based on AICc
Isothermality	bio3	No, not significant based on AICc
Temperature Seasonality	bio4	No, highly correlated with bio11
Max Temp of Warmest Month	bio5	No, highly correlated with bio11
Min Temp of Coldest Month	bio6	No, highly correlated with bio11
Temperature Annual Range	bio7	No, highly correlated with bio11
Mean Tempe of Wettest Quarter	bio8	No, not significant based on AICc
Mean Temp of Driest Quarter	bio9	No, highly correlated with bio11
Mean Temp of Warmest Quarter	bio10	No, highly correlated with bio11
**Mean Temp of Coldest Quarter**	**bio11**	**Yes**
Annual Precipitation	bio12	No, highly correlated with bio17 and bio19
Precipitation of Wettest Month	bio13	No, not significant based on AICc
Precipitation of Driest Month	bio14	No, highly correlated with bio17 and bio19
Precipitation Seasonality	bio15	No, not significant based on AICc
**Precipitation of Wettest Quarter**	**bio16**	**Yes**
**Precipitation of Driest Quarter**	**bio17**	**Yes**
Precipitation of Warmest Quarter	bio18	No, not significant based on AICc
Precipitation of Coldest Quarter	bio19	No, poorer geographic goodness-of -fit
**January precipitation**	**jan_pcp**	**Yes**
**Land use/cover**	**LUC**	**Yes**
Elevation	alt	No, not significant based on AICc

#### Mosquito collections

*Aedes albopictus* adults were collected in three states (Pennsylvania, New Jersey, New York) covering most of the known geographic range of this species in northeastern USA. Collections were conducted using mostly CDC miniature light traps and gravid traps supplemented by other methods (aspiration, mosquito magnet, BG Sentinel traps, Zumba trap) during 2001–2011 in Pennsylvania, 2002–2011 in New Jersey, and in 2004 (first detection)-2011 in Suffolk County, Long Island, New York. The surveillance database contained a total of 11,632 *Ae. albopictus* presence records, with 5,361 unique geographic locations (Supplemental Table S1). For all locations, *Ae. albopictus* presence in the traps during any period of time was mapped to the native WorldClim 30 arcsec (approximately 1×1 km) grid. Additional known locales where this species has been collected in New York City and its northern and eastern suburbs, southern Connecticut, and isolated southern New England areas were not included in model development, but were useful for model validation.

### Statistical Modeling

Statistical modeling was conducted using Maxent v3.3.3 k, a machine learning algorithm for modeling species distribution estimated from the presence data-only and from the environmental variables [27], [28]. Thus, it is especially suited for mosquito surveillance since these records typically represent a reliable presence, but only an unreliable absence data for a particular mosquito species. In addition to modeling current species distribution, Maxent has built-in capabilities to predict the future range by using two sets of environmental variables using the MESS analysis tool [28]. Current environmental conditions generate the model, and a set of altered environmental variables is then used to project the future changes.

Compared to other available algorithms, Maxent performance consistently ranked among the best [29]. However, when used to predict areas climatically suitable for invasion by non-native species, Maxent was found to be overly sensitive to the choice of modeling parameters with model over-fitting, multicollinearity, and data-dredging (i.e using large number of environmental layers) negatively affecting the prediction's accuracy [30]. To address these statistical issues, a model selection procedure based on Akaike information criterion (AICc) was proposed [30], [31]. Comparative analysis of different models generated by Maxent was done using ENMTools v1.3 software [32].

The modeling for this study was conducted in two steps. The first model was created with a small number of *a priori* defined and best fitted climatic variables to avoid data-dredging [30]. Specifically, winter temperature and precipitation were shown as the most critical climatic factors limiting *Ae. albopictus* abundance and distribution in northeastern USA and other areas close to its northernmost boundary distribution [21], [23]. Landscape variables were then entered in the model and retained if the goodness-of-fit was improved. Elevation was selected because it defines different climatic conditions and provides physical barriers to dispersion. Land use/cover (LUC) was selected because *Ae. albopictus* reaches the highest densities in urbanized environments in northeastern USA [21].

Minimum convex polygons (MCP, [30]) were used to define the region of *Ae. albopictus* presence encompassing the most of the current geographic distribution of the species with good surveillance coverage (Figure 1). Coordinate-based locations enabled fine geographic scale of the analysis at the highest resolution (30 arcsec or approx. 1×1 km), which corresponded to the limited flight range (<1 km) of *Ae. albopictus* [33]. The MCP MaxEnt model was run 25 times, withholding a different 10% of the localities each time to estimate the parameters and the precision. The model was then projected into the baseline and the three future climatic conditions (2020s, 2050s, and 2080s) to identify areas suitable for *Ae. albopictus*. Model overfitting protection (i.e., increased regularization parameter [30]) were explored using AICc. Default MaxEntauto feature setting (linear, quadratic, product, threshold and hinge) were used.

## Results

### MCP model selection

WorldClim temperature variables (bio1-bio11; Table 1) were highly correlated with each other (|r|≥0.86, ENMTools) with the exception of bio2, bio3, and bio8. To avoid multicollinearity and data-dredging, bio2, bio3, bio8 and bio11 (see Table 1 for details) were selected for inclusion in the model based on the strength of association and previous research [20], [21], [23]. Precipitation variables (Table 1) were highly intercorrelated (|r|≥0.85, ENMTools). Bio15, bio16, bio17, bio18, bio19, and jan_pcp (see Table 1 for details) were included in the initial model given the importance of winter precipitation (i.e., snow cover) and precipitation regularity (i.e., dry/wet periods) [23], [34]. Model selection using AICc (ENMTools) resulted in the best-fitted model containing bio11 and bio19 and the second best model containing bio11, bio16, bio17, and jan_pcp. Entering elevation did not contribute any additional information to either model, while entering LUC significantly improved the goodness-of-fit for both models. While the model containing bio11, bio19, and LUC had the best goodness-of-fit (AICc = 64500.47), it did not predict suitable *Ae. albopictus* habitat along the southern New England coast when projected into current climatic conditions (data not shown). The second best model (AICc = 65189.36) containing bio11 (mean temp of coldest quarter), bio16 (precipitation of wettest quarter), bio17 (precipitation of driest quarter), jan_pcp (January precipitation), and LUC had a better geographic fit to known *Ae. albopictus* range in northeastern USA and was thus selected as final.

The final MCP model had AUC_test_ = 0.919 indicating very good model performance and the omission rate (proportion of test points not predicted) = 0.012, which was significantly better than random prediction at p<0.001 by binomial test. Mean temperature of coldest quarter was the most significant environmental factor defining the current range of *Ae. albopictus* (85.0% importance, higher temperatures more suitable). Mean temperatures below -2.0°C had near zero probability of *Ae. albopictus* presence, while those between 0°C and +1.0°C had the highest probabilities. LUC contributed 9.6% of the information in the model, with response dependent on each category. Developed urban areas were the most likely to support *Ae. albopictus* presence (probability  = 0.65), while forested areas and open agricultural areas were the least likely (probability = 0.10 and 0.12, respectively). Combined precipitation contribution to the model was 5.4%. January precipitation (3.0%, higher precipitation more suitable) was more important followed by precipitation of driest quarter (2.0%, higher precipitation more suitable) and precipitation of wettest quarter (0.4%, lower precipitation more suitable).

### Current and future Ae. albopictus range in northeastern USA

**Figure 2:**
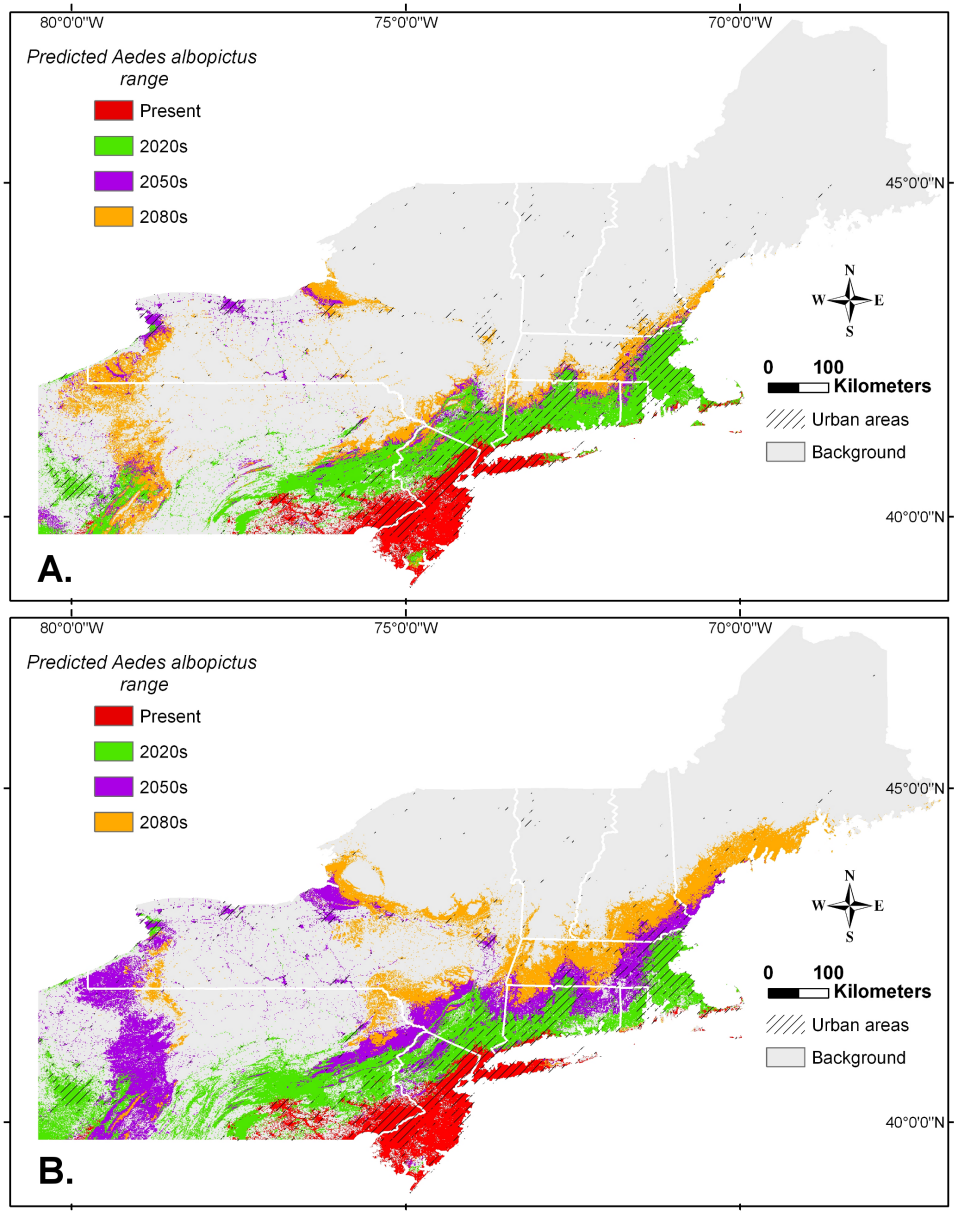
Predicted *Ae. albopictus* range expansion in the northeastern USA under two climate change scenarios. (**A**) Moderate increase in CO2 emissions (B2 scenario). (**B**) Higher increases in CO_2_ emissions (A2 scenario). Predicted present range based on 1950–2000 climate data. Three future time periods: 2020s (years 2010–2039), 2050s (years 2040–2069), and 2080s (years 2070–2099). Urban areas are indicated (2010 US Census Bureau).

The MCP model was projected into current and future climatic conditions (Figure 2). The lowest *Ae. albopictus* presence threshold to predict and map the areas suitable for this species was set at the equal sensitivity and specificity (probability_presence_ = 0.29). This value was very similar to the probability_presence_ = 0.31,which included 90% of mapped *Ae. albopictus* occurrence records, and was close to one half of the maximum *Ae. albopictus* presence probability value of 0.65 calculated by the MaxEnt model. Using the threshold probability_presence_ = 0.29, the model identified the current range suitable for *Ae. albopictus* closely corresponding to known surveillance records from southeastern Pennsylvania through southern and central New Jersey, New York City and Long Island (Figure 2). Smaller suitable areas were identified along the southern Connecticut coast and isolated areas in coastal Rhode Island and Massachusetts. The model performed less well in western Pennsylvania, identifying smaller suitable areas than that suggested by the existing surveillance records. Overall, about 5% of the total area in the Northeast was classified as suitable. Among major urban areas, most of the greater New York City metropolitan area (pop. 12.2 M) with the exception of northern suburbs, Philadelphia metropolitan area (pop. 3.8 M), Harrisburg and Lancaster, PA (pop. 850,000), Trenton, NJ (pop. 300,000), and Atlantic City, NJ (pop. 250,000) are currently within the *Ae. albopictus* suitable range.

Under both B2 and A2 CO_2_ emission scenarios, significant expansions of the current *Ae. albopictus* range was predicted for the period of 2010–2039 (Figure 2A,B: 2020s). The suitable range territory would increase by the factor of three from 5% to 16% of the entire northeastern USA under both scenarios. The most expansion would occur in southern New England where this species was predicted to occupy extensive areas in Connecticut, Rhode Island, and eastern Massachusetts including major urban centers of Boston metropolitan area (pop. 4.1 M), Harford-Waterbury, CT (pop. 1.1 M), Providence, RI (pop. 930,000), Bridgeport-Stamford, CT (pop. 880,000), New Haven, CT (pop. 560,000), and Barnstable Town, MA (pop. 250,000). In New York, New Jersey, and Pennsylvania the suitable conditions would expand north to northwest, to include the entire metropolitan New York City, Pittsburgh metropolitan area, PA (pop. 1.7 M.), and Allentown (pop. 630,000), Scranton, PA (pop. 380,000). Suitable habitat would also exist along the south shore of Lake Erie in Erie, PA (pop. 200,000) and in parts of Buffalo, NY (pop. 936,000).

The models' outputs for the middle to last parts of the century were somewhat different in spatial extent, but showed similar trends of decelerating rates of expansion. The moderate CO_2_ emissions model B2 predicted the suitable *Ae. albopictus* range expanding to 27% of the total area in 2040–2069 (Fig. 2A: 2050s), most notably along the coast of Lake Erie and Ontario into New York's urban centers of Buffalo, Rochester (pop. 720,000), and Syracuse (pop. 412,000). Suitable conditions would further expand in 2070–2099 to include 43% of the total area of northeastern USA, especially in rural western Pennsylvania and New York (Fig. 2A: 2080s). In New England, *Ae. albopictus* range was predicted to extend into New Hampshire (Dover-Rochester-Portsmouth, pop. 150,000) and southern Maine (Portland, pop. 204,000) along the coast, and more inland in Connecticut and Massachusetts (Springfield, pop. 531,000; Worcester, pop. 453,000), and New York (Albany, pop. 595,000; Binghamton, pop. 155,000).

Under higher CO_2_ emissions model A2, similar changes would occur but at a more rapid pace. *Aedes albopictus* range under A2 model would expand to 32% of the total area of northeastern USA by the middle of this century, 2040–2069 (Fig. 2B: 2050s). Many areas characterized as suitable under A2 model in 2050s were also predicted to become suitable under B2 model, but later on in 2080s (Fig. 2A: 2080s). The suitable climatic conditions would exist in extensive areas in western Pennsylvania and New York, along the coast of the Great Lakes in Buffalo, Rochester, Syracuse, and in lower Hudson valley including Albany. In New England, the range will expand in eastern and central Massachusetts, and coastal New Hampshire into coastal southern Maine. Under A2 model in 2070–2099, suitable range would occupy almost one-half (49%) of the total Northeast area extending into most of Massachusetts and the southernmost parts of Vermont, further inland in New Hampshire and extensively in coastal Maine. In New York, further expansion will occur in Hudson and Mohawk valleys. Thus, under A2 model, *Ae. albopictus* is predicted to occur in all major urban centers and in all but the northernmost urban areas in northeastern USA by the end of the 21^st^ century.

## Discussion

### Modeling Aedes albopictus current and future range in northeastern USA

Climatic factors likely represent the major constraints on the extent of *Ae. albopictus* expansion northward [35]. A number of studies have confirmed the inability of diapausing *Ae. albopictus* eggs to survive extreme cold temperatures in the winter. In laboratory, temperate *Ae. albopictus* eggs' long-term survival threshold was close to -12°C in North America [19] and −10°C in Europe [22] for up to 24 hr exposure time. These thresholds were below the −5°C January isotherm first used to model *Ae. albopictus* distribution in North America based on the native temperate Asian localities [20]. The discrepancy is not surprising since *Ae. albopictus*' niche shifted in the invaded regions making predictions employing the original range less accurate [24]. In our study, mean winter temperature resulted in slightly better model goodness-of fit than minimum coldest month (January) temperature, with which it was highly correlated (Pearson's r = 0.99). Switching mean winter with minimum January temperature displayed null presence probability below −9°C (roughly corresponding to −2°C mean winter temperature), close to the thresholds identified under laboratory conditions. Winter temperature was the most crucial factor in this study accounting for 85% of the model similarly to the previous study of *Ae. albopictus* populations in New Jersey where winter temperature explained about 99% of the variability in the adult production [21].

Despite its importance, winter temperature might not be sufficient to define the range of *Ae. albopictus* in its entirety. Under field conditions, the correlation between winter temperature and *Ae. albopictus* egg survival was not linear, being moderated by the snow cover that insulated the eggs and allowed successful overwintering at much lower temperatures [23]. In our study, snow cover was assumed to be correlated with the amount of January precipitation that mostly falls as snow in northeastern USA. Inclusion of January precipitation in the model improved the geographic goodness-of-fit indicating increased *Ae. albopictus* presence probability peaking at 70 to 90 mm range and then declining again likely due to extreme cold conditions in the areas with heavy snowfall. January precipitation performed better than winter (i.e. coldest quarter) precipitation to increase the geographic fit of the model to the known *Ae. albopictus* range, especially in the marginal areas such as New England and southwestern Pennsylvania. One possible explanation for this is that the coldest quarter precipitation might not provide a good snow cover estimate in many parts of northeastern USA, where rain or mixed precipitation may predominate during warmer winter months. In addition to snow cover, another potentially important climatic factor is precipitation variability, with increased drying inducing higher *Ae. albopictus* mortalities [34]. This factor was demonstrated especially significant in warmer subtropical climates, but less so under more temperate conditions. In agreement with the results of their study, the contribution of variability (i.e. amount of precipitation during the driest and the wettest quarters) in our model was low; nevertheless the overall geographic fit of the model was improved when those two variables were included.

Apart from climatic factors, landscape features also play an important role in mosquito distributions, especially weak fliers with very short dispersal distances such as *Ae. albopictus* [33]. While elevation was not significant when entered in the model, likely because it was accounted for by other variables, land use was the second most important variable. Specifically, three categories (urban areas, forested areas, and open agricultural areas) showed strong positive or negative associations with *Ae. albopictus* presence. Urban areas increased the probability of presence, which was in agreement with a previous study demonstrating strong *Ae. albopictus* affinity to urbanized environments in northeastern USA [21]. Forested areas might be less susceptible to *Ae. albopictus* invasions, whereas agricultural areas are mostly open crop or pasture fields with little protective cover and lack of container habitats for *Ae. albopictus* larval production. Urban areas with the surrounding suburban envelopes were, therefore, considered the future “hotspots” of high *Ae. albopictus* activity if located within the predicted range. It is difficult to make predictions on future changes in urban environments, which were held constant at the 2010 level for the modeling purposes. However, these changes will likely occur within the already existing urban environs, and thus remain largely incorporated in our models.

Two recent global modeling studies included predictions of the current *Ae. albopictus* range extent in North America. The first by Benedict et al. [3] employed a different algorithm (GARP) using a different dataset of 11 environmental layers at coarser spatial resolution making direct comparison between the models difficult. However, it appears that the current *Ae. albopictus* suitable habitat in northeastern USA identified by Benedict et al. [3] was overrepresented, being more similar to the range predicted by the middle of this century (i.e. 2050s) in our models. Specifically, most of Connecticut and eastern Massachusetts, as well as the areas along the Great Lakes were classified as highly suitable under the GARP model, but do not provide current presence records to support these predictions. Our current model predicted a much smaller range in New England (coastal southern Connecticut and Massachusetts) where *Ae. albopictus* has been detected repeatedly over the last few years. Those discrepancies might be due to coarser resolution of the GARP model, lower accuracy of GARP generated models compared to those by Maxent [29], and differences in underlying environmental variables.

The second global modeling study by Medley [24] used Maxent with a similar set of environmental variables, resulting in *Ae. albopictus* range estimates in northeastern USA closer to our model, albeit at coarser spatial resolution. Similarly to our model, the extent of the current *Ae. albopictus* range in southwestern Pennsylvania was underestimated, suggesting additional variables not captured by the models as important for delineating suitable *Ae. albopictus* habitat in that area. It is possible that repeated summer reintroductions from the mid-Atlantic states immediately to the south of this region is mostly responsible for these discrepancies between the predicted established range and the actual range reflecting the leading edge of *Ae. albopictus* expansion. Similarly to Benedict et al. [3] but different from our model, Medley [24] overestimated the current *Ae. albopictus* range in New England. One reason for this difference might have been Maxent sensitivity to multicollinearity [30], which was not captured by Medley [24], but specifically addressed in our study by using a more stringent AIC-based selection procedure for the environmental layers inclusion in the model.

Our study differed from both Benedict et al. [3] and Medley [24] in using (a) precise geographic locations where *Ae. albopictus* was collected as opposed to the county centroids, (b) minimum convex polygons [30] to delineate the areas of *Ae. albopictus* presence more accurately, (c) information criterion (AIC) based model and variable selection process, and (d) sampling points from the same region to create a model that inherently provides more accurate estimates at a regional level [36]. We then projected the current model into the future climate change scenarios and identified winter temperature as the most crucial factor in the model. Incidentally, warming winters are the most significant outcomes of climate change in northeastern USA, far exceeding other potential changes such as warming summers or increases in winter precipitation [25].

Rising winter temperatures will drive the expansion of *Ae. albopictus'* suitable range from the current 5% (approximately 36,000 sq. km) to about 16% (107,000 sq. km) of the total northeastern USA area in the next three decades, regardless of the climatic model used. The proportion of people residing in urban areas most susceptible to high *Ae. albopictus* levels [21] will increase from the current 32% of the total population to just over one-half (about 53%). After the rapid range expansion period in the coming decades, the rate is expected to slow becoming more gradual under both models. The estimates vary from 27% (B2) to 32% (A2) of the total area of northeastern USA in the 2050s, and from 43% (B2) to 49% (A2) by the end of the century. Under both models, most major urban areas of northeastern USA situated in coastal areas (either along the Atlantic or the Great Lakes) will support suitable climate for *Ae. albopictus* by the middle of this century. Afterwards, the bulk of the range expansion is expected occur into more rural areas. Western Pennsylvania is a good example where a combination of increased winter temperatures and significant snow cover during the coldest part of the year might create climatic conditions suitable for *Ae. albopictus* establishment. However, the rural environment of this area is unlikely to support significant *Ae. albopictus* populations. By the end of the century, almost one-half of the northeastern USA with all major urban areas containing over 60% of the total population will be suitable for *Ae. albopictus*, further exacerbating resources of vector control officials tasked with protection of public health and comfort.

### Implications for public health practitioners

**Figure 3:**
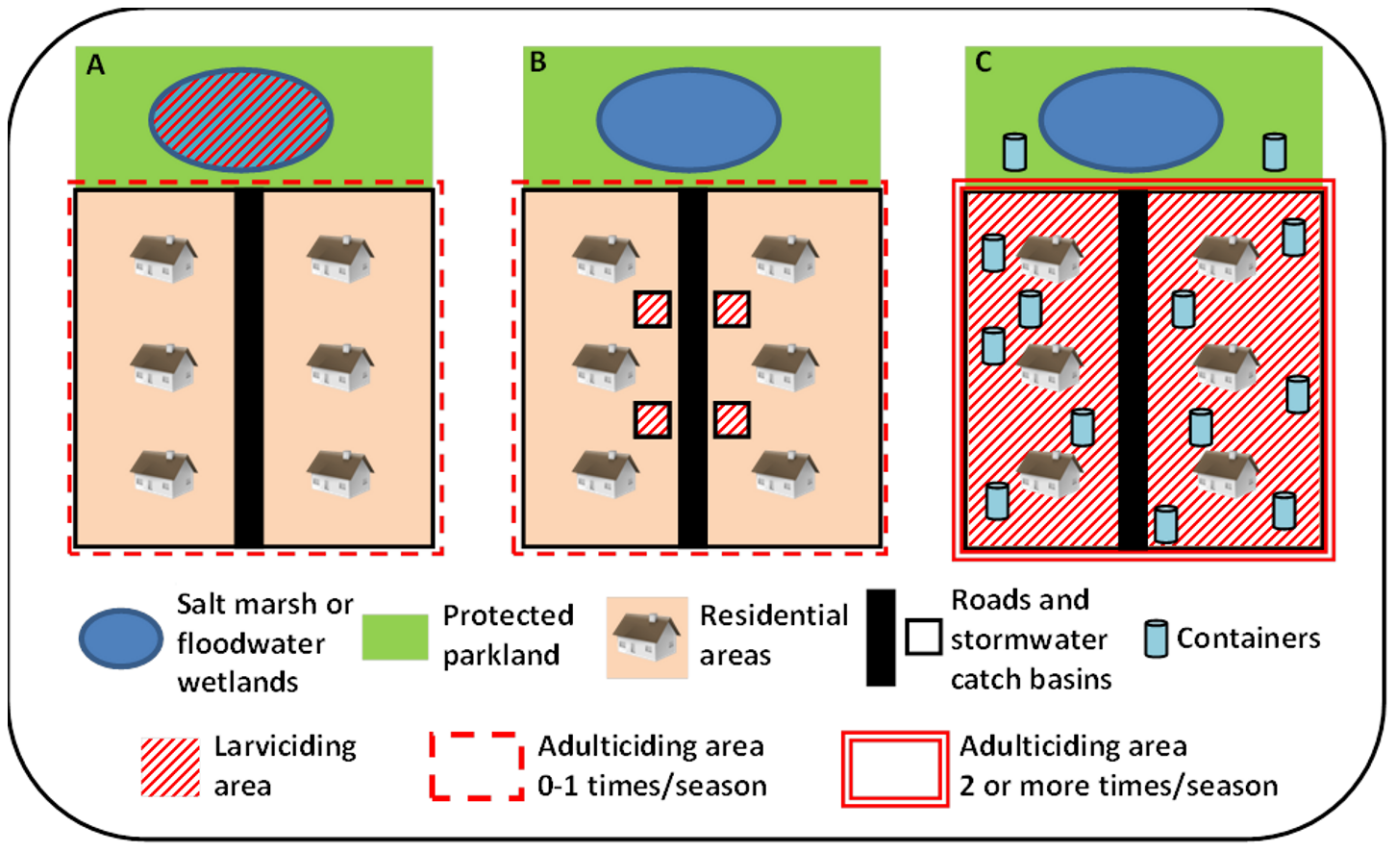
Schematic representation of mosquito habitat and control paradigm. (**A**) Salt marsh and floodwater *Aedes* spp. emerge outside of residential areas and may be effectively controlled at the larval wetland habitat with timely larvicide applications (no adulticiding required). Heavy infestation may require infrequent (usually once per season) adulticide applications. (**B**) Urban *Culex* spp. utilize manmade stormwater structures within the residential areas. Delayed release larvicide formulations are very effective in suppressing *Culex* spp. emergence. In the years with high WNV activity, a timely adulticide application may be required for prevention of virus transmission to humans. (**C**) *Ae. albopictus* is a container-inhabiting species whose larval habitat is unpredictable and widespread throughout the residential as well as the adjacent natural areas. Effective control of biting adults may require combined areawide larvicide and adulticide applications, likely to be repeated multiple times during a mosquito season.

The Northeastern region has a population of over 55 million people (about 18% of the USA total), and contains some of the country's major metropolitan areas such as those associated with New York City, Philadelphia and Boston. Before the arrival of WNV in New York City in1999, mosquito-borne diseases were not an important concern in Northeastern metropolitan areas or even in most suburbs, and relatively little attention was paid to mosquito control. For instance, in 1999, New York City had no organized mosquito control program and the initial response to the virus outbreak required acquisition of outside experts and pesticide application contractors [37]. Historically, mosquito control programs were first established in coastal areas in response to biting problems caused by salt marsh species [38]. Also of concern were inland areas that required mosquito control to combat nuisance *Aedes* spp. from floodwater habitats as well as vectors of eastern equine encephalitis from various freshwater habitats [39] (Figure 3A). With the introduction of WNV, mosquito control programs were upgraded in many areas to focus on the primary vectors (*Culex* spp.) important in the transmission cycle of this pathogen, thereby shifting the emphasis to “urban” habitats high in organic material such as wastewater treatment facilities and stormwater catch basins (Figure 3B). While these habitats are numerous, they are also easily delineated and remain largely unchanged from year to year even in highly dynamic tidal wetlands [40]. The response, therefore, can be planned accordingly and efficaciously following environmental conditions such as rains, floods, and tides. Alternatively, known urban larval habitats such as catch basins can be treated with well-developed methods providing long term relief for the entire mosquito season.

Compared to these more traditional mosquito control habitats, the rise of *Ae. albopictus* presents a very different and difficult set of problems for public health practitioners in the northeastern USA (Figure 3C). Unlike mosquito species traditionally encountered in the Northeast, *Ae. albopictus* larvae prefer small, artificial container habitats which are ubiquitous and diffusely distributed in urban areas and nearby parkland. Additionally, many of these container habitats are located within private residential backyards that might be inaccessible to mosquito control personnel. A problem area is often not identified until the impacted residents are overwhelmed and begin requesting service. Surveillance efforts are made difficult by the large quantity of potential habitats as well as their ephemeral nature, increasing and decreasing on a continual basis. When sources (containers) are found, they must be abated or treated one by one, and even when they are eliminated, new ones frequently appear [41].

If larval control becomes impractical in an area, adulticiding may be required. However, effective adulticiding of *Ae. albopictus*, is difficult at best and may require multiple applications to be effective [42]. While new methods of dispersing larvicides through areawide truck-mounted methods can make control of larvae less labor-intensive, the entire community must be treated, compared to small targeted sites typical of floodwater *Aedes* spp. or urban *Culex* spp.; adulticiding is still likely to be required (Figure 3). Combined with the need to treat a larger portion of the community than is the case for saltmarsh and floodwater mosquitoes, or urban *Culex* spp., both surveillance and control of *Ae. albopictus* are more labor intensive and accrue significantly higher costs.

Measures to control this species, therefore, can easily outstrip the resources available to mosquito control programs, especially since they are already stretched to deal with their traditional problem set. The problems faced by local public health agencies in many urban and rural areas of northeastern USA with no organized mosquito control programs will be even more challenging. *Ae. albopictus* range expansion in the Northeast threatens to present challenges far exceeding the resources likely to be available to combat them unless new and effective control strategies are developed. A key factor in determining the success of these strategies will be whether they can be implemented at reasonable cost to very large areas. Until they are developed, more and more communities in the Northeast will have to adapt to the presence of this species in significant numbers.

Anticipating areas of potential establishment while planning ahead and gathering sufficient resources will be the key for successful public health campaigns. A broad effort in community sanitation and education at all levels of government and the private sector is required. It may be appropriate to increase the role of private pest control operators offering mosquito control services to provide barrier treatments or other specialized and localized control that is currently beyond the means of public entities. The groundwork for possible large-scale adulticiding needs to be implemented as well, both in terms of identifying resources and putting plans in place to determine under what circumstances such control would be initiated. None of this will be easy, but unless improved strategies are developed to prevent infestations of *Ae. albopictus*, these measures will be necessary on an increasing scale in the near future.

## Conclusions

The Asian tiger mosquito, *Ae. albopictus*, is poised to significantly expand its range in the northeastern United States in the next few decades primarily due to warming winter temperatures. By the end of the 21^st^ century, the climatic conditions suitable for *Ae. albopictus* will exist to cover roughly one-half of the land area in the northeastern USA. More than 30 million people, especially those in urbanized environments, will reside within the Asian tiger mosquito range, and will be potentially subjected to high biting populations of this species and impending arboviral threats. Currently, there are no cost effective options for control of *Ae.albopictus*. Thus, its range expansion will present serious challenges to the local public health agencies, particularly in the areas with weak or non-existent mosquito abatement infrastructure. Better planning and improved control methods will be the key to dealing with this public health threat.

## Supporting Information

Table S1 ***Aedes albopictus*** **collection locations.**
(XLS)
